# Supplementary material for: A convenient polyculture system that controls a shrimp viral disease with a high transmission rate
Source: Commun Biol. 2021 Nov 11;4:1276. doi: 10.1038/s42003-021-02800-z (PMC8585955; doi:10.1038/s42003-021-02800-z)
Supplement: Supplementary file 2 — Supplementary Information [file 42003_2021_2800_MOESM2_ESM.pdf]

Supplementary Information for

A convenient polyculture system that controls a shrimp viral disease with a high transmission rate

Supplementary Note

Polyculture system of coculturing Pacific white shrimp (*Litopenaeus vannamei*) and grass carp (*Ctenopharyngodon idella*)

1. Salinity < 6 ‰
2. Stocking density of shrimp postlarvae:
   Earthen pond: 4.5×10^5 ~ 7.5×10^5/ha
3. Introduce grass carp 20~30 days after releasing shrimp postlarvae in the pond
4. Stocking density of grass carp: 450 ~ 900/ha
5. Body weight of grass carp: 0.5 ~ 1.0 kg
6. Once diseased shrimp appear in the pond, stop feeding the shrimps for at least 3 days. Shrimp feeding can resume after the diseased shrimp disappear.

Polyculture system of coculturing Pacific white shrimp (*Litopenaeus vannamei*) and African sharptooth catfish (*Clarias gariepinus*)

1. Salinity < 6 ‰
2. Stocking density of shrimp postlarvae:
   Earthen pond: 4.5×10^5 ~ 7.5×10^5/ha
3. Introduce African sharptooth catfish 10 days after releasing shrimp postlarvae in the pond
4. Stocking density of African sharptooth catfish: 600 ~ 900/ha
5. Body weight of African sharptooth catfish: 0.15 ~ 0.5 kg
6. Once diseased shrimp appear in the pond, stop feeding the shrimps for at least 3 days. Shrimp feeding can resume after the diseased shrimp disappear.

Polyculture system of coculturing black tiger shrimp (*Penaeus monodon*) and brown-marbled grouper (*Epinephelus fuscoguttatus*)

1. Salinity: 13~35 ‰
2. Stocking density of shrimp postlarvae:
   Earthen pond: 4.5×10^5 ~ 7.5×10^5/ha
3. Introduce brown-marbled grouper 10~15 days after releasing shrimp postlarvae in the pond
4. Stocking density of brown-marbled grouper: 900 ~ 3,000/ha
5. Body weight of brown-marbled grouper: 0.1 kg
6. Once diseased shrimp appear in the pond, stop feeding the shrimp for at least 3 days. Shrimp feeding can resume after the diseased shrimp disappear.

Polyculture system of coculturing kuruma shrimp (*Marsupenaeus japonica*) and branded goby (*Chaeturichthys stigmatias*)

Cultivation condition:

1. Salinity > 15 ‰
2. Stocking density of shrimp postlarvae:
   Earthen pond: < 1.8×10^5/ha
3. Introduce branded gobies 20 days after releasing shrimp postlarvae in the pond
4. Stocking density of branded goby: 750 ~ 900/ha
5. Body weight of branded goby: 0.05 kg
6. Once diseased shrimp appear in the pond, stop feeding the shrimp for at least 3 days. Shrimp feeding can resume when the diseased shrimp disappear.

Polyculture system of coculturing Chinese white shrimp (*Fenneropenaeus chinensis*) and branded goby (*Chaeturichthys stigmatias*)

Cultivation condition:

7. Salinity > 15 ‰
8. Stocking density of shrimp postlarvae: < 1.8×10^5/ha
9. Introduce branded gobies 20 days after releasing shrimp postlarvae in the pond
10. Stocking density of branded goby: 750 ~ 900/ha
11. Body weight of branded goby: 0.05 kg
12. Once diseased shrimp appear in the pond, stop feeding the shrimp for at least 3 days. Shrimp feeding can resume when the diseased shrimp disappear.

Supplementary Figures

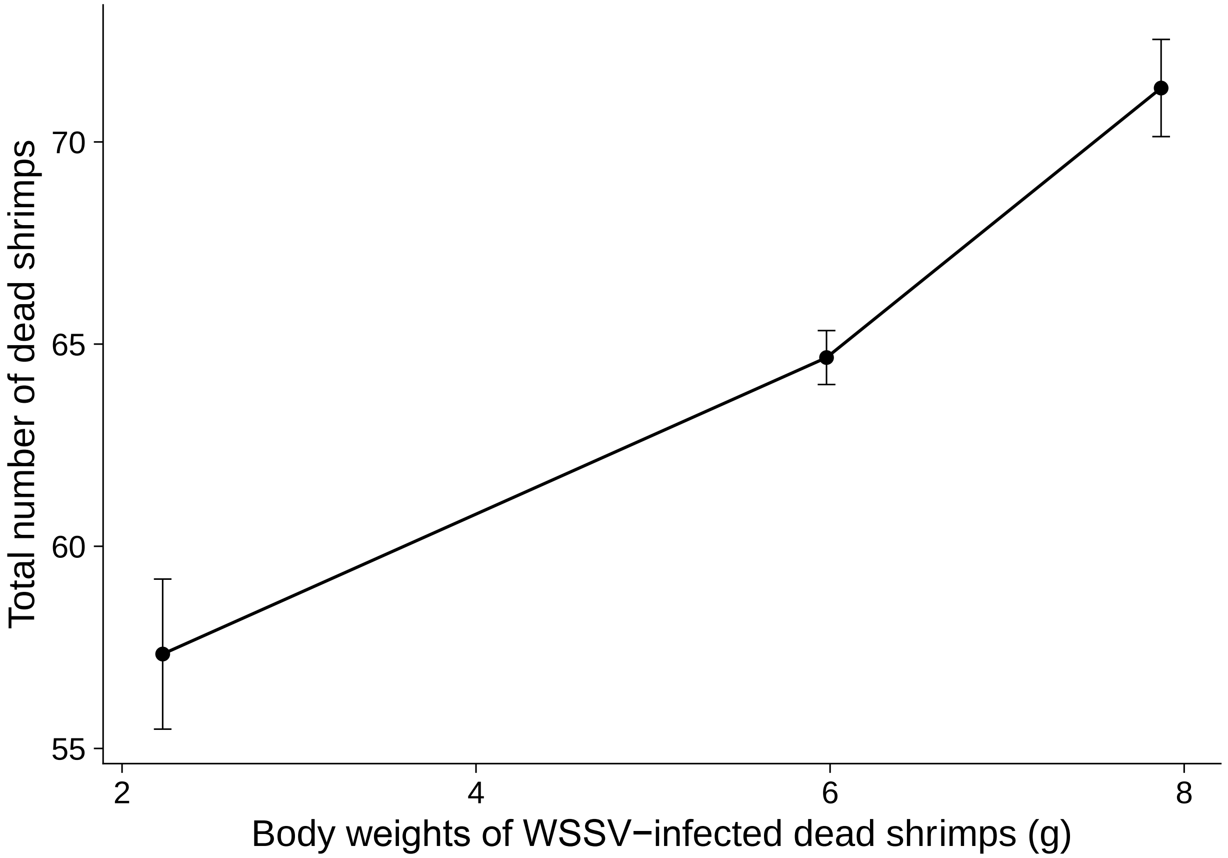

Supplementary Figure 1 | Number of healthy shrimp died from WSSV infection in aquarium where one piece of dead WSSV-infected shrimp of body weight of 1.98 g, 6.13 g, and 7.95 g, respectively, was added. Means and standard errors are shown. With the initial dead WSSV-infected shrimps become heavier, the basic reproduction number (R_0) of WSSV increases.

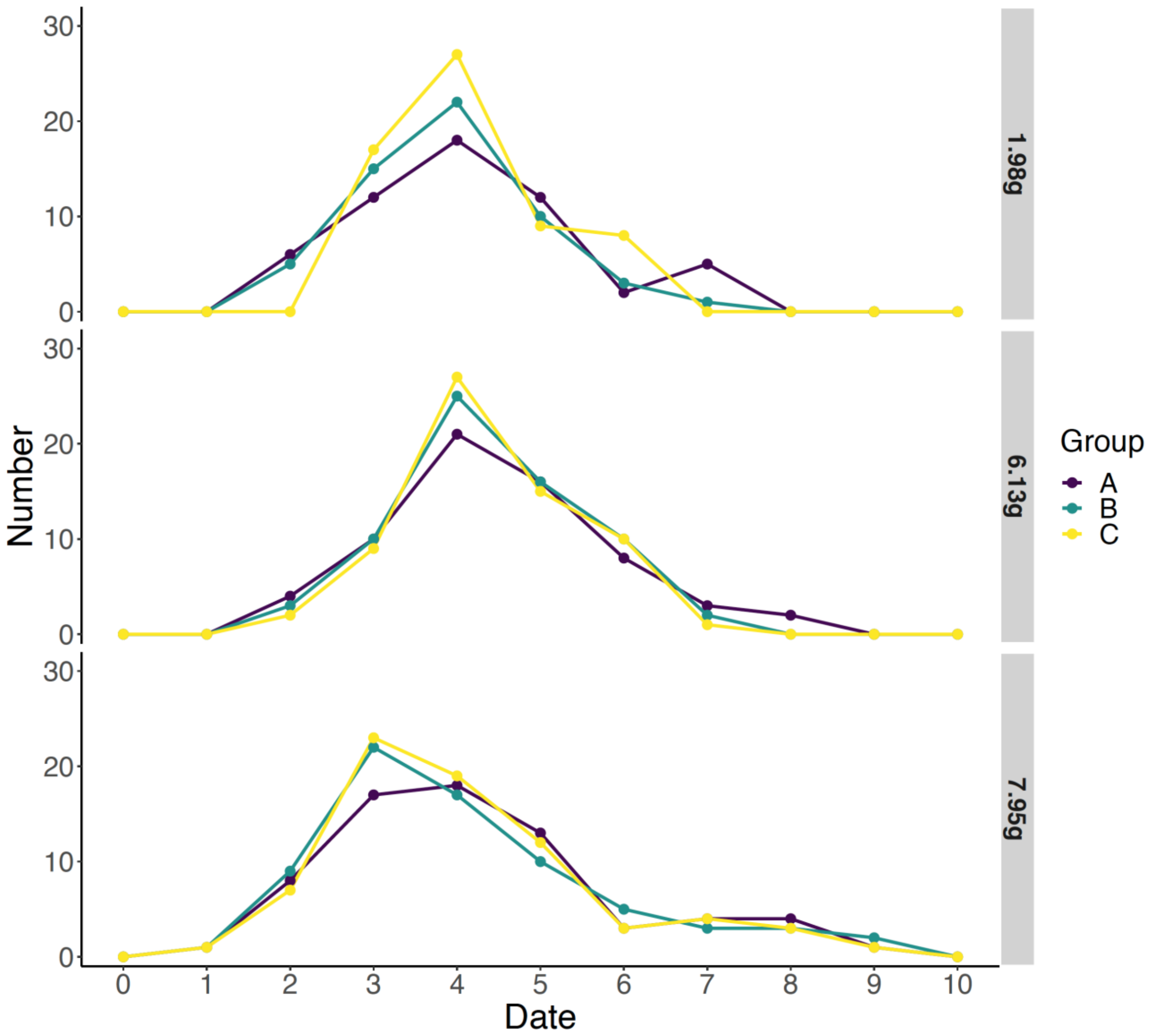

Supplementary Figure 2 | Daily mortality number of healthy shrimps in aquarium where one piece of dead WSSV-infected shrimp of body weights of 1.98 g, 6.13 g, and 7.95 g, respectively, was added. Time to death was consistent across the three groups of body weight for WSSV-infected shrimp, with the majority on the third to sixth day and the peak number of deaths on the fourth and fifth days.

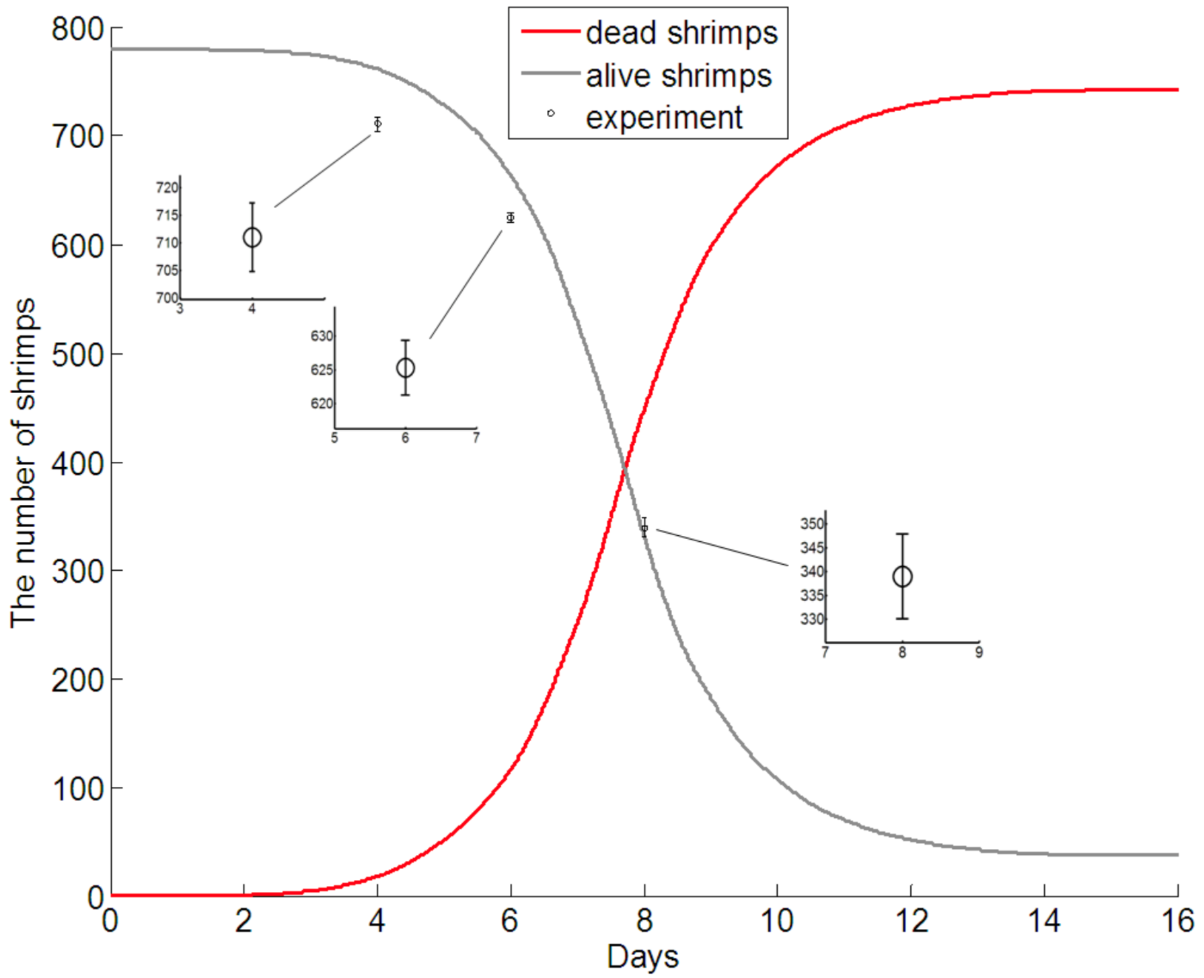

Supplementary Figure 3 | The quantity of dead shrimps (red) and live shrimps (grey) (include healthy shrimps and infected shrimps) concerning time. The red curve and the grey curve are the results of the simulation. The three small open circles (dots) with error bars were the numbers of live shrimps on the fourth, sixth, eighth day from the artificial infection experiments. This data set is derived from three repeats of independent experiments, which is expressed as mean ± SD. The experimental results were consistent with the results of mathematical modeling.

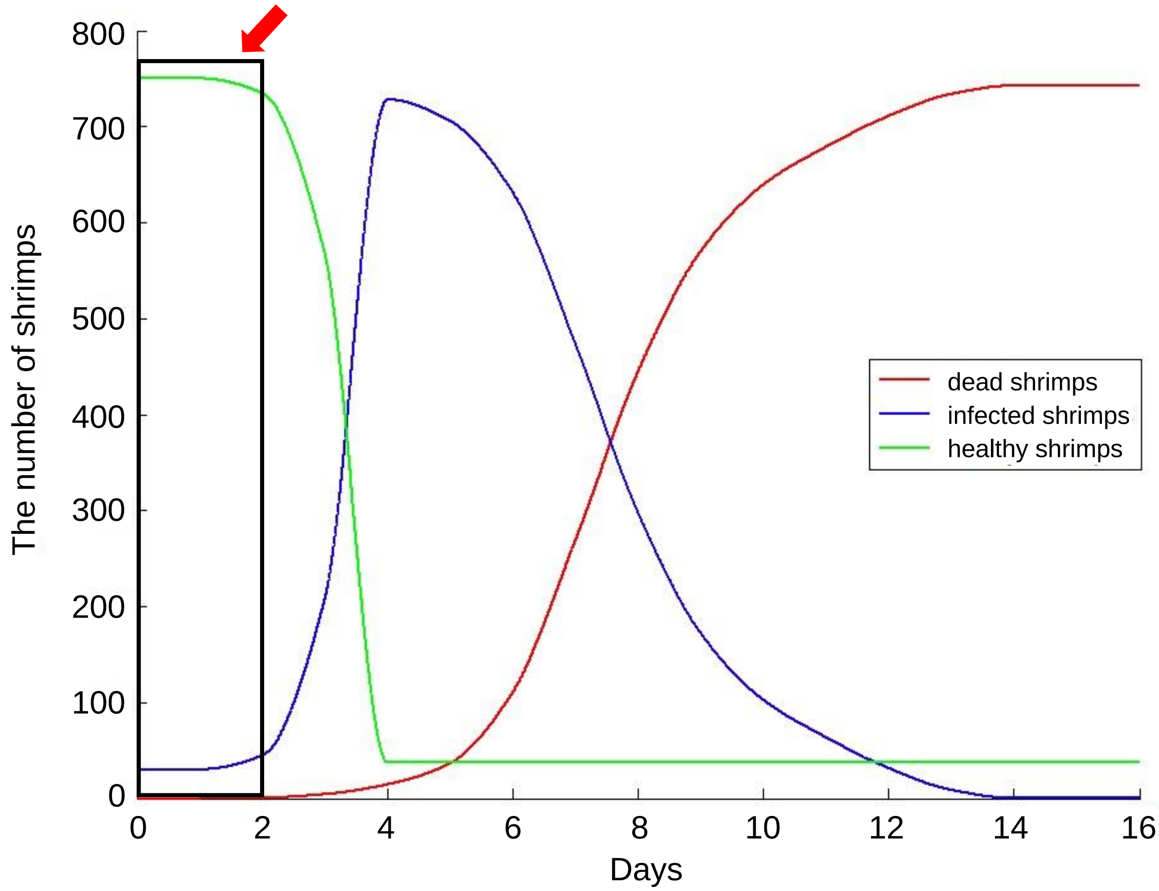

Supplementary Figure 4 | The changes of number of healthy, WSSV-infected, and dead shrimps over time. The numbers of healthy shrimp (green), infected shrimp (blue) and dead shrimp (red) were derived from Model 2. The number of live shrimp began to decrease 2 days after WSSV infection and drastically decreased 4 days after WSSV infection. The number of infected shrimp drastically increased 2 days after WSSV infection. The number of infected shrimp began to decrease 4 days after infection, as infected shrimp became dead shrimp. This led to the increment of dead shrimp 6 days after infection. Finally, all shrimp died except for the ones that might resistant to WSSV infection. The time window for WSS prevention is highlighted in grey box.

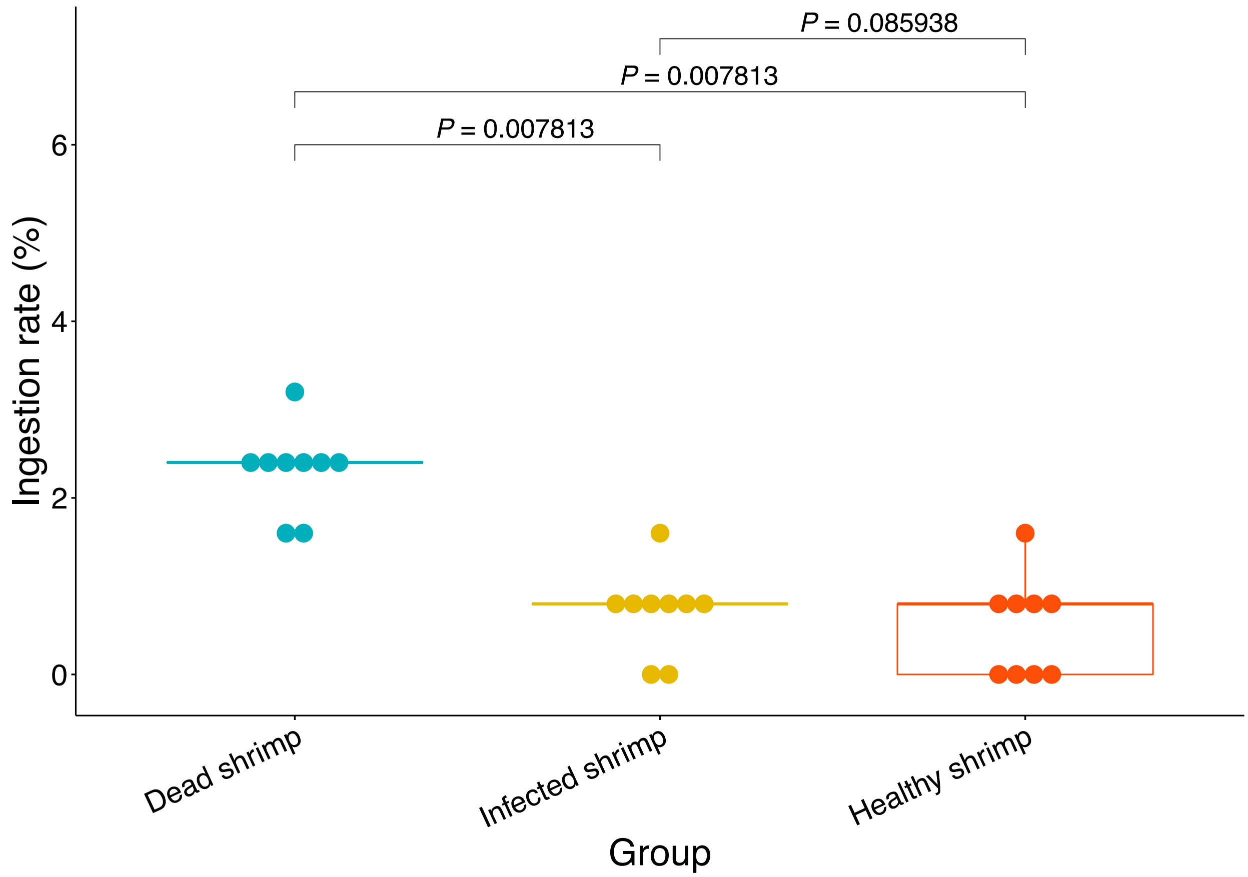

Supplementary Figure 5 | Feeding selectivity of African sharptooth catfish on dead, infected (endopod and exopod removed), and healthy shrimps. The diseased shrimp infected with WSSV died within two days, which makes it hard to distinguish the initial dead shrimp from the ones that were died from diseased shrimp. The diseased shrimp had reduced activity, and the activity of shrimp was reduced after the endopods and exopods were removed. Thus, the shrimp with endopods and exopods removed were utilized to resemble WSSV-infected shrimp. *P*-values (permutation test, paired) were labeled (n=9). African sharptooth catfish ingested significantly more dead shrimp than infected (endopod and exopod removed) and healthy shrimp.

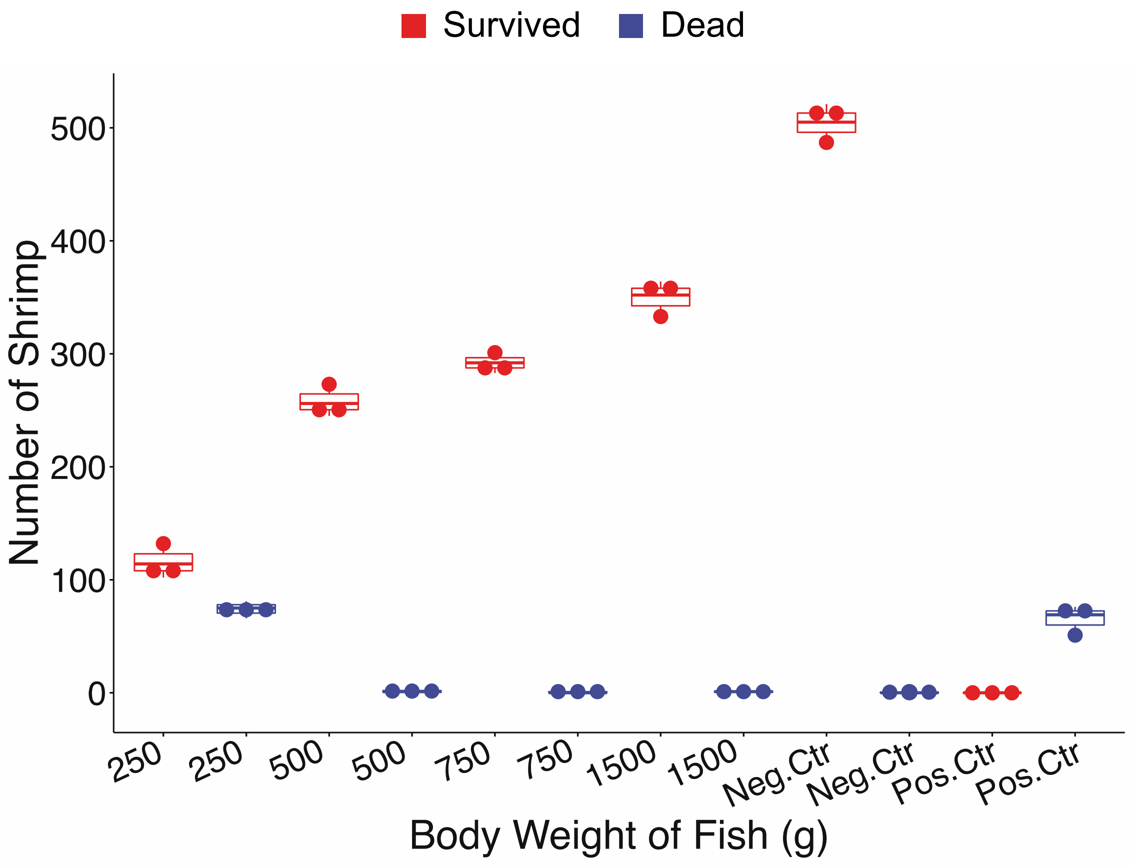

Supplementary Figure 6 | The effect of the different body weights of African sharptooth catfish on the control of WSS outbreaks. Experimental ponds were set up in which 600 healthy and WSSV-carrying shrimp and 3 WSSV-infected shrimp with the same body weights were cultured with one African sharptooth catfish of different body weights. After 14 days in culture, the ponds cocultured with one catfish weighing 0.25kg, 0.5 kg, 0.75 kg, and 1.5 kg showed shrimp survival rates of 19%, 40.83%, 48.67%, and 55.5%, respectively. Nearly all dead shrimp were removed by fish in the ponds cocultured with catfish of bodyweights greater than 0.5 kg. This suggests the suitable body weight of co-cultured catfish to control WSS outbreak is 0.5kg. Furthermore, co-culturing of shrimp and fish can control the WSS outbreaks even when there are WSSV carriers in shrimp postlarva.

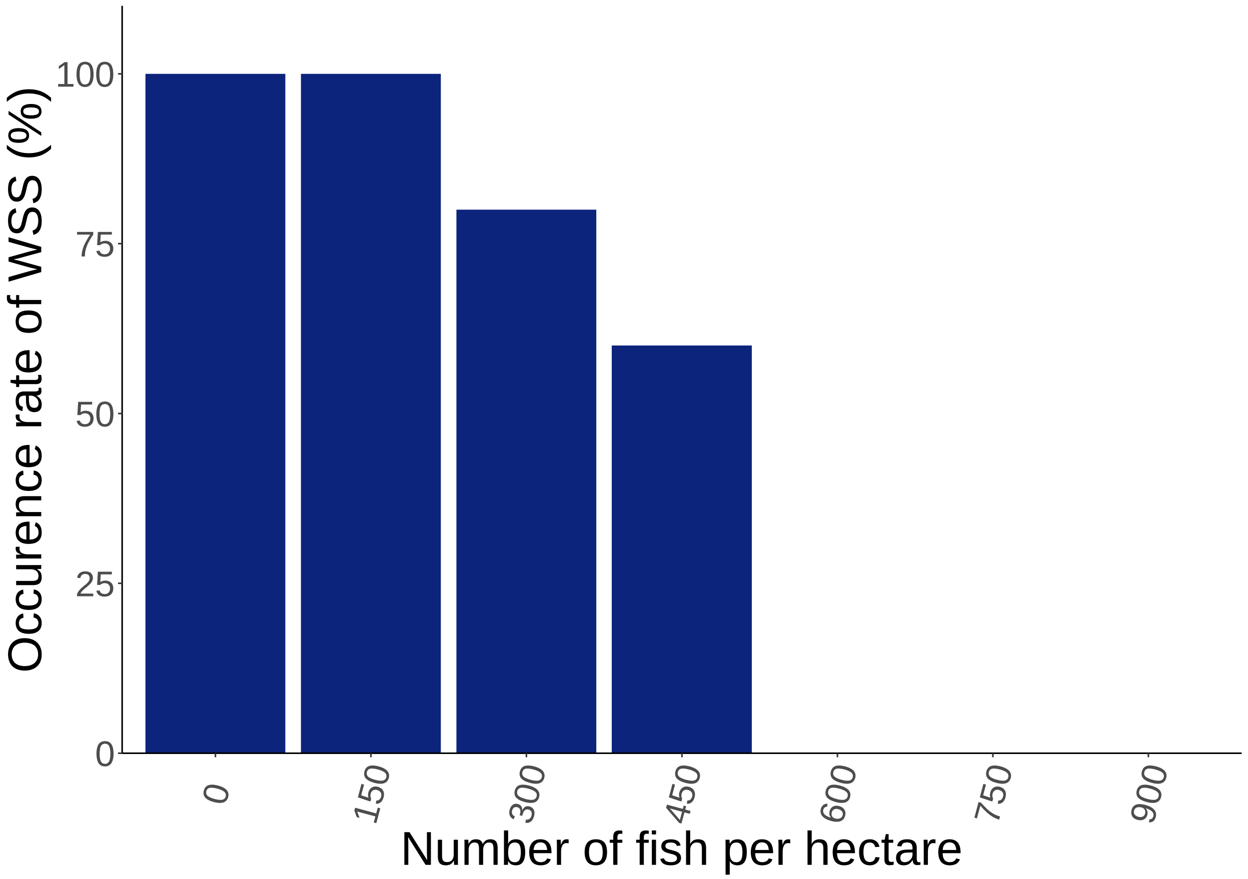

Supplementary Fig 7 | The relationship of the number of cocultured African sharptooth catfish and the occurrence rate of WSS. More than 600 catfish of approximately 0.5 kg per hectare can completely control the outbreaks of WSS, but fewer than 450 catfish cannot fully control outbreaks of WSS.

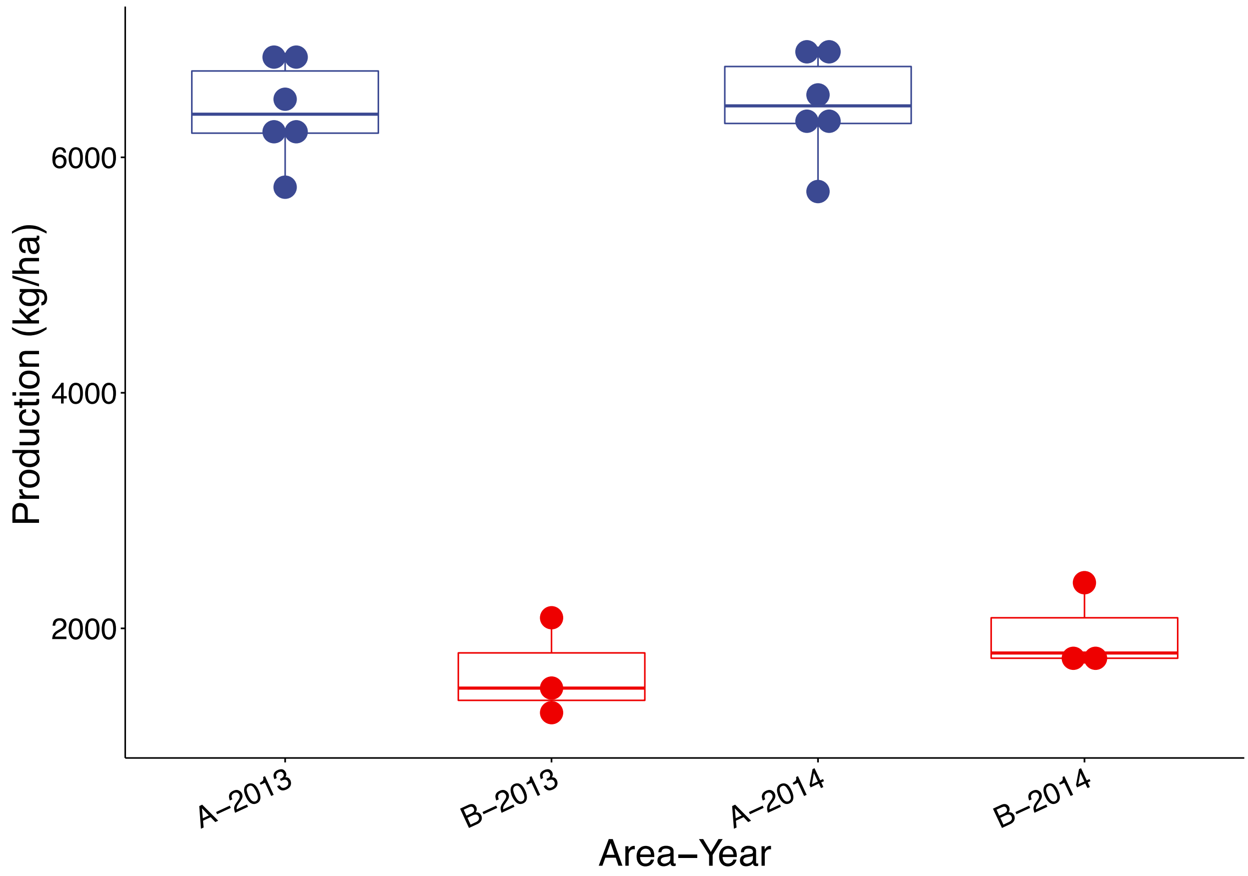

Supplementary Figure 8 | Total yield of *Penaeus monodon* production in ponds with (red) or without (blue) brown-marbled grouper (*Epinephelus fuscoguttatus*) at a farm in Changjiang, Hainan Province, China (Farm 3). Releasing brown-marbled grouper substantially increased shrimp production.

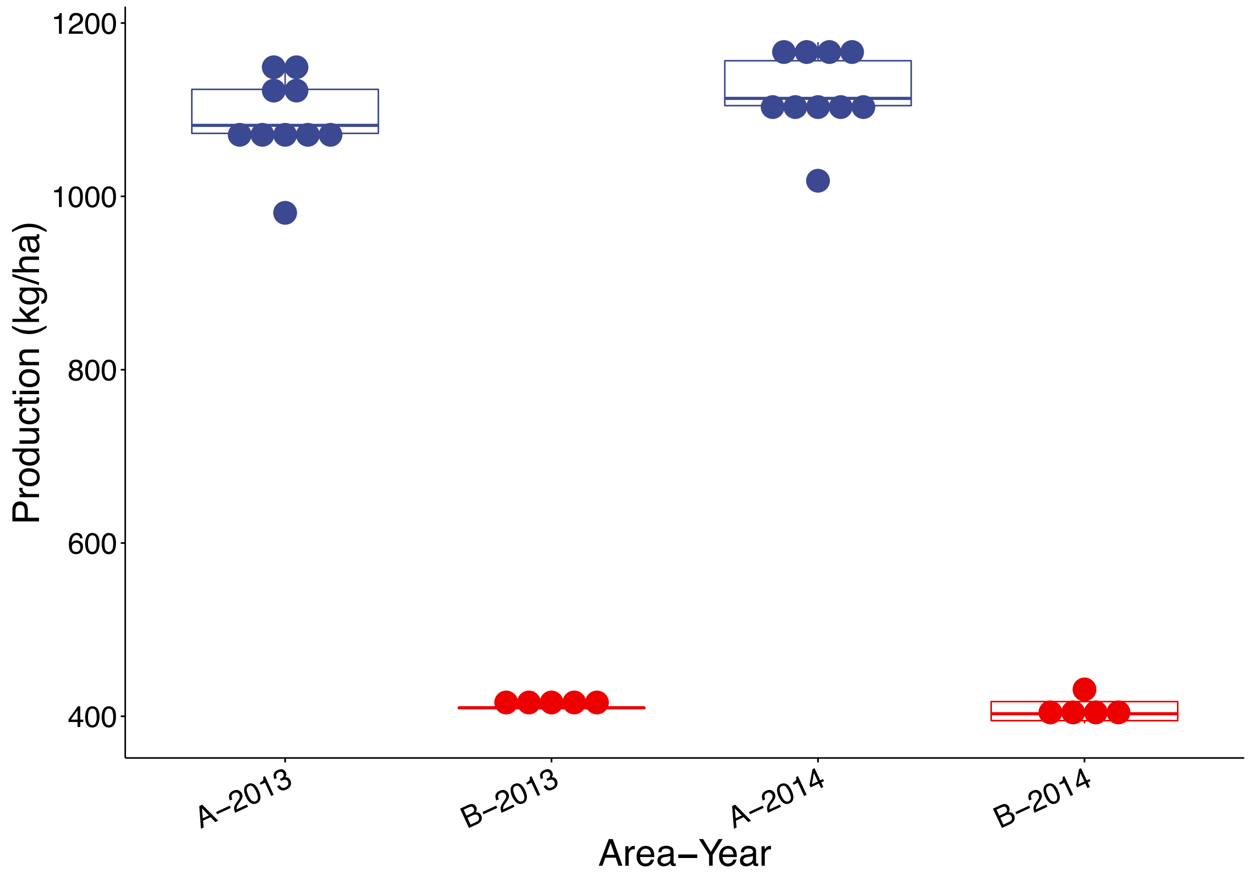

Supplementary Figure 9 | Total yield of *Marsupenaeus japonica* production in ponds with (red) or without (blue) branded goby (*Chaeturichthys stigmatias*) at a farm in Qingdao, Shandong Province, China (Farm 4). Means and standard errors are shown. Releasing brown-marbled grouper substantially increased shrimp production.

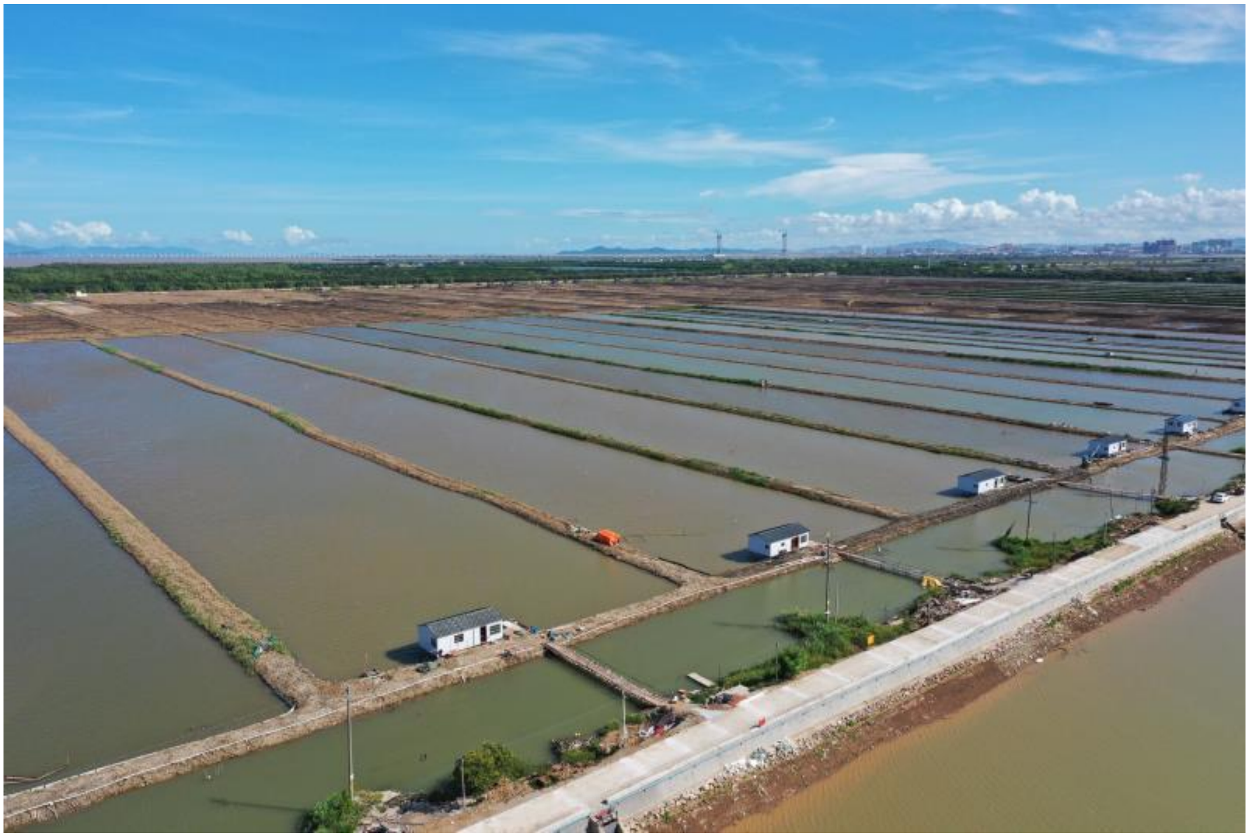

Supplementary Figure 10 | The farms' association in Nansha, Guangdong Province, China. Each farmer has an earthen pond (3 ha) to cultivate shrimp. Photo taken by Xinjian Liu.

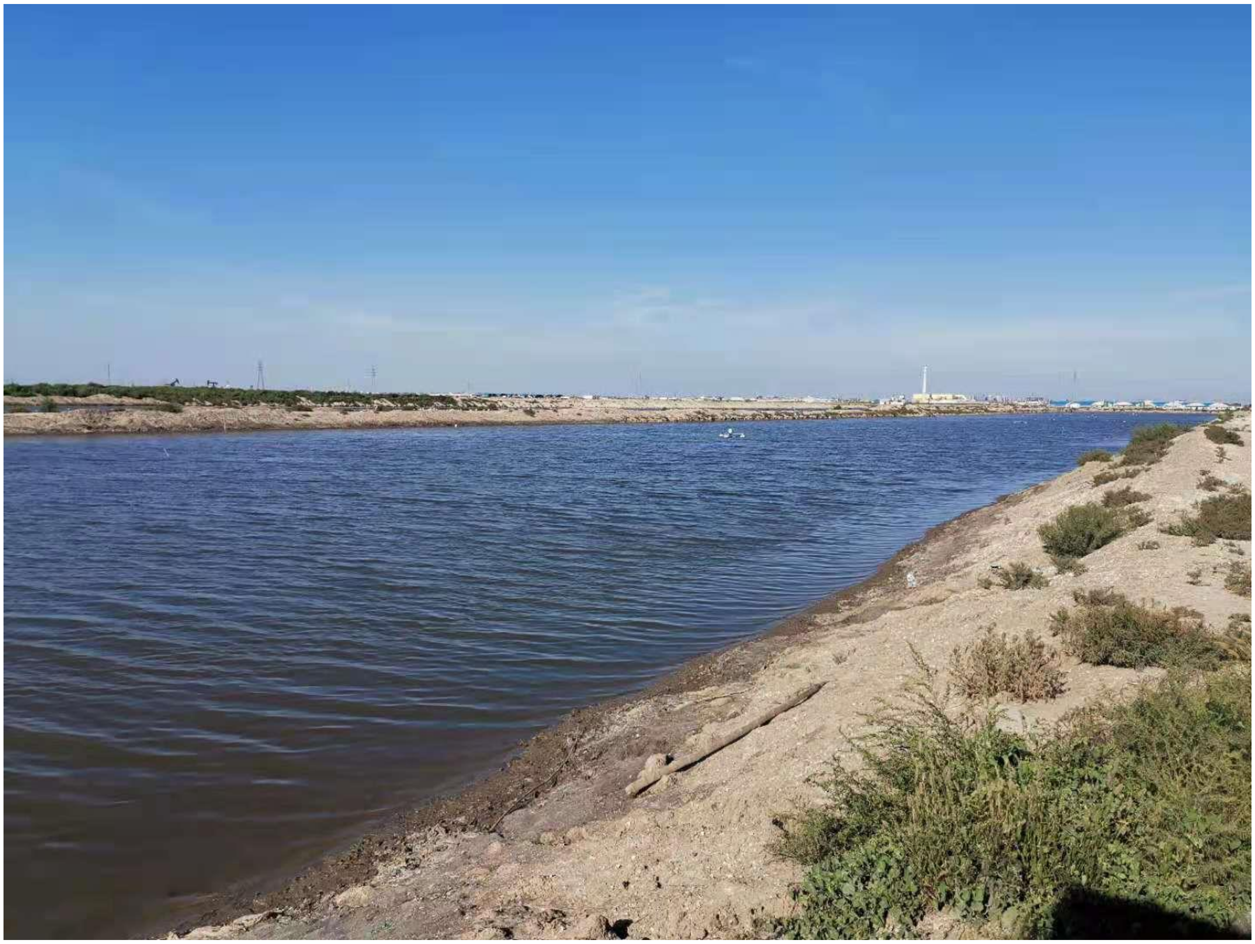

Supplementary Figure 11 | The pond at the farms' association in Tanghai, Hebei Province, China. Each farmer has an earthen pond (5 ha) to cultivate shrimp. Photo taken by Muhua Wang.

Supplementary Tables

Supplementary Table 1. Number of healthy shrimp died from WSSV infection in aquarium where one piece of dead WSSV-infected shrimp of body weight of 1.98 g, 6.13 g, and 7.95 g, respectively, was added.

	1.98 g	6.13 g	7.95 g
Group A	55	64	69
Group B	56	66	72
Group C	61	64	73
Mean	57.3	64.7	71.3
Negative Control	1	0	2

Supplementary Table 2. Daily mortality of healthy shrimps in aquarium where one piece of dead WSSV-infected shrimp weighting 1.98 g was added

Date	Aquarium A	Aquarium B	Aquarium C	Negative Control
**0**	0	0	0	0
**1**	0	0	0	0
**2**	6	5	0	0
**3**	12	15	17	1
**4**	18	22	27	0
**5**	12	10	9	0
**6**	2	3	8	0
**7**	5	1	0	0
**8**	0	0	0	0
**9**	0	0	0	0
**10**	0	0	0	0

Supplementary Table 3. Daily mortality of healthy shrimps in aquarium where one piece of dead WSSV-infected shrimp weighting 6.13 g was added

Date	Aquarium A	Aquarium B	Aquarium C	Negative Control
**0**	0	0	0	0
**1**	0	0	0	0
**2**	4	3	2	0
**3**	10	10	9	0
**4**	21	25	27	0
**5**	16	16	15	0
**6**	8	10	10	0
**7**	3	2	1	0
**8**	2	0	0	0
**9**	0	0	0	0
**10**	0	0	0	0
**11**	0	0	0	0

Supplementary Table 4. Daily mortality of healthy shrimps in aquarium where one piece of dead WSSV-infected shrimp weighting 7.95 g was added.

Date	Aquarium A	Aquarium B	Aquarium C	Negative Control
**0**	0	0	0	0
**1**	1	1	1	0
**2**	8	9	7	0
**3**	17	22	23	0
**4**	18	17	19	0
**5**	13	10	12	0
**6**	3	5	3	1
**7**	4	3	4	0
**8**	4	3	3	0
**9**	1	2	1	1
**10**	0	0	0	0
**11**	0	0	0	0
**12**	0	0	0	0

Supplementary Table 5. The change of survived shrimp number during WSSV transmission

	Pond 1	Pond 2	Pond 3
Day 2	712	717	705

	Pond 4	Pond 5	Pond 6
Day 4	626	629	622

	Pond 7	Pond 8	Pond 9
Day 8	339	331	348

Note: Nine cement ponds (315cm × 315cm × 120cm) were set up with water volume of 5 m^3 and salinity of 8‰. Regarding the stocking quantity of 7.5×10^5/ha in shrimp farming production, 750 healthy shrimp with an average body weight of 7.9 g were cultured in each of the nine ponds. Healthy shrimp were quarantined for seven days before the experiment started. Thirty artificially WSSV-infected shrimp were then introduced in each pond. The numbers of survived shrimp were counted in three ponds on the 2nd, 4th, 8th day after WSSV infection, respectively.

Supplementary Table 6. Daily dead shrimp ingestion of grass carp (*Ctenopharyngodon idellus*) with body weight ranges from 0.5 kg to 1.5 kg

Group	0.5 kg	1 kg	1.5 kg
	Pond A (1.643 kg)	Pond B (3.011 kg)	Pond C (5.270 kg)
Day 1	9.53%	8.53%	7.17%
Day 2	9.72%	8.16%	7.30%
Day 3	9.13%	8.88%	7.10%
Day 4	8.88%	7.84%	7.15%
Day 5	9.09%	8.14%	7.28%
Mean	9.27%	8.31%	7.20%

Note: The shrimp ingestion rate of fish is quantified by the daily ingestion rate (weight of ingested shrimps per day / fish weight). The total body weight of fish in each pond is listed under the pond name. The mean body weight of dead shrimp used in the experiment is 5.3 g.

Supplementary Table 7. Daily dead shrimp ingestion of African sharptooth catfish (*Clarias gariepinus*) with body weights ranges from 0.262 kg to 1.502 kg

	Pond A (0.262 kg)	Pond B (0.496 kg)	Pond C (0.731 kg)	Pond D (1.502 kg)
Day 1	7.2%	6.0%	4.8%	4.4%
Day 2	9.6%	6.0%	4.8%	4.0%
Day 3	7.2%	6.0%	4.0%	4.4%
Day 4	7.2%	6.0%	5.6%	4.8%
Day 5	7.2%	4.8%	4.8%	4.4%
Mean	7.68%	5.76%	4.8%	4.4%

Note: The body weight of fishes in each pond is listed under the pond name. The shrimp ingestion rate of fish is quantified by the daily ingestion rate (weight of shrimps ingested by fish per day / fish body weight). The mean body weight of dead shrimps used in the experiment is 6.2 g.

Supplementary Table 8. Daily dead shrimp ingestion of red drum (*Sciaenops ocellatus*) with body weight ranges from 0.590 kg to 0.732 kg

	Pond C (0.590 kg)	Pond B (0.654 kg)	Pond A (0.732 kg)
Day 1	9.33%	9.85%	10.13%
Day 2	12.67%	12.31%	10.13%
Day 3	11.33%	12.92%	12.27%
Day 4	14.00%	13.54%	11.73%
Day 5	12.67%	9.23%	12.27%
Mean	12.00%	11.57%	11.31%

Note: The body weight of fishes in each pond is listed under the pond name. The shrimp ingestion rate of fish is quantified by the daily feeding rate (weight of shrimps ingested by fish per day / fish body weight). The mean body weight of dead shrimps used in the experiment is 3.9 g.

Supplementary Table 9. Daily healthy shrimp ingestion of grass carp (*Ctenopharyngodon idellus*) of body weight around 1 kg

	Pond A	Pond B	Pond C	Pond D (Negative Control)
Fish weight	1.050 kg	0.956 kg	1.013 kg	n/a
Number of healthy shrimps	750	750	750	750
Number of healthy shrimps reduced	63	61	67	24
Number of healthy shrimps ingested	39	37	43	n/a
Number of healthy shrimps ingested (daily)	3.9	3.7	4.3	n/a
Body weight of healthy shrimps ingested (daily)	20.67g	19.61g	22.79g	n/a
Daily ingestion rate of healthy shrimps	1.97%	2.05%	2.25%	n/a

Note: Three experimental ponds and one control pond (315cm × 315cm × 120cm) were set up with water volume of 5 m^3 and salinity of 5‰. In total, 750 healthy shrimp with an average bodyweight of 5.3 g were cultured in each pond. One grass carp weighting 0.956 kg, 1.013 kg, and 1.050 kg was released in each of the experiment ponds, respectively. No fish was released in the control pond. Every two days, 50% of the water in each pond was changed. Live shrimp remained in each pond were counted and weighted after 10 days of the experiment. The shrimp ingestion rate of fish is quantified by the daily ingestion rate (weight of shrimps ingested by fish per day / fish body weight). The number of healthy shrimp ingested by the fish is calculated by subtracting the number of healthy shrimp reduced in Pond D (negative control) from the number of healthy shrimp reduced in each of the experimental ponds.

Supplementary Table 10. Daily healthy shrimp ingestion of African sharptooth catfish (*Clarias gariepinus*) of body weight around 1 kg

	Pond A	Pond B (Negative Control)
Fish weight	1050g	n/a
Number of healthy shrimps	300	300
Number of healthy shrimps reduced	45	21
Number of healthy shrimps ingested	24	n/a
Number of healthy shrimps ingested (daily)	4.8	n/a
Body weight of healthy shrimps ingested (daily)	10.56g	n/a
Daily ingestion rate of healthy shrimps	1.01%	n/a

Note: One experimental pond and one control pond (315cm × 315cm × 120cm) were set up with water volume of 5 m^3 and salinity of 3‰. In total, 750 healthy shrimp with an average bodyweight of 2.2 g were cultured in each pond. One African sharptooth fish weighting 1.050 kg was released in the experiment pond. No fish was released in the control pond. The shrimp ingestion rate of fish is quantified by the daily ingestion rate (weight of shrimps ingested by fish per day / fish body weight). The number of healthy shrimps ingested by the fish is calculated by subtracting the number of healthy shrimps reduced in Pond B (negative control) from the number of healthy shrimps reduced in the experimental pond.

Supplementary Table 11. Daily healthy shrimp ingestion of red drum (*Sciaenops ocellatus*) of body weights around 0.5 kg

	Pond A	Pond B	Pond C	Pond D (Negative Control)
Fish weight	0.519 kg	0.554 kg	0.595 kg	n/a
Number of healthy shrimps	750	750	750	750
Number of healthy shrimps remained	618	648	596	702
Number of healthy shrimps reduced	132	102	154	48
Number of healthy shrimps ingested	84	54	106	n/a
Number of healthy shrimps ingested (daily)	12	7.7	15.1	n/a
Body weight of healthy shrimps ingested (daily)	32.4g	20.79g	40.77g	n/a
Daily ingestion rate of healthy shrimps	6.24%	4.01%	7.86%	n/a

Note: Three experimental ponds and one control pond (315cm × 315cm × 120cm) were set up with water volume of 5 m^3 and salinity of 5‰. In total, 750 healthy shrimp with an average bodyweight of 2.7 g were introduced in each pond. One red drum weighting 0.519 kg, 0.554 kg, and 0.595 kg was released in each of the experiment ponds, respectively. No fish was released in the control pond. The shrimp ingestion rate of fish is quantified by the daily ingestion rate (weight of shrimps ingested by fish per day / fish body weight). The number of healthy shrimps ingested by the fish is calculated by subtracting the number of healthy shrimps reduced in Pond D (negative control) from the number of healthy shrimps reduced in each of the experimental ponds.

Supplementary Table 12. Feeding selectivity of grass carp (*Ctenopharyngodon idella*) on healthy, infected (endopod and exopod removed) and dead shrimp

	Dead Shrimps	Infected (endopod and exopod removed) Shrimps	Healthy shrimps
Day 1	5.1%	1.1%	0. 9%
Day 2	4.9%	1.3%	0.2%
Day 3	5.1%	1.1%	0.7%
Day 4	4.7%	1.1%	0.7%
Day 5	4.9%	1.3%	0.9%
Day 6	5.1%	2%	0
Day 7	5.3%	1.6%	0.2%
Day 8	5.5%	1.6%	0.4%
Day 9	4.9%	1.3%	0.4%
Mean	5.1%	1.4%	0.5%

Note: The diseased shrimp infected with WSSV died within two days, which makes it hard to distinguish the initial dead shrimp from the ones that were died from diseased shrimp. The diseased shrimp had reduced activity, and the activity of shrimp was reduced after the endopods and exopods were removed. Thus, the shrimp with endopods and exopods removed were utilized to resemble WSSV-infected shrimp. The shrimp ingestion rate of fish is quantified by the daily ingestion rate (weight of shrimps ingested by fish per day / fish body weight). The body weight of grass carp is 1.58 kg. The mean weight of shrimp used in the experiment is 3.5 g.

Supplementary Table 13. Feeding selectivity of African sharptooth catfish (*Clarias gariepinus*) on healthy, infected (endopod and exopod removed) and dead shrimp

	Dead Shrimps	Infected (endopod and exopod removed) Shrimps	Healthy shrimps
Day 1	2.4%	0.8%	1.6%
Day 2	2.4%	0.8%	0.8%
Day 3	3.2%	1.6%	0%
Day 4	2.4%	0.8%	0%
Day 5	2.4%	0.8%	0.8%
Day 6	1.6%	0.8%	0.8%
Day 7	2.4%	0	0
Day 8	2.4%	0.8%	0
Day 9	1.6%	0	0.8%
Mean	2.31%	0.71%	0.53%

Note: The diseased shrimp infected with WSSV died within two days, which makes it hard to distinguish the initial dead shrimp from the ones that were died from diseased shrimp. The diseased shrimp had reduced activity, and the activity of shrimp was reduced after the endopods and exopods were removed. Thus, the shrimp with endopods and exopods removed were utilized to resemble WSSV-infected shrimp. The shrimp ingestion rate of fish is quantified by the daily ingestion rate (weight of shrimps ingested by fish per day / fish body weight). The body weight of African sharptooth catfish is 1.03 kg. The mean weight of shrimp used in the experiment is 8.4 g.

Supplementary Table 14. Suitable body weights of grass carp that are capable of controlling WSS outbreaks

Body weight (kg)	Number of shrimps	Number of live shrimps in 13 days of post-infection	Rate of survival (%)
0.3	600	0	0
	600	0	0
	600	0	0
0.5	600	0	0
	600	0	0
	600	0	0
1	600	493	82
	600	486	81
	600	501	84
1.5	600	460	77
	600	467	78
	600	453	76
Negative control	600	502	84
	600	511	85
	600	493	82
Positive control	600	0	0
	600	0	0
	600	0	0

Supplementary Table 15. Suitable body weights of African sharptooth catfish that are capable of controlling WSS outbreak determined by experiments

Body weight (kg)	Number of shrimps	Number of live shrimps in 14 days of post-infection	Rate of survival (%)	Number of dead shrimps remained in the pond
0.25	600	114	19	75
	600	132	22	81
	600	102	17	66
0.50	600	245	41	1
	600	256	43	3
	600	273	46	0
0.75	600	283	47	0
	600	292	49	2
	600	301	50	0
1.50	600	352	59	1
	600	333	56	2
	600	364	61	0
Negative control	600	487	81	0
	600	521	87	0
	600	505	84	0
Positive control	600	0	0	76
	600	0	0	69
	600	0	0	51

Note: Experimental ponds were set up in which 600 healthy and WSSV-carrying shrimp and 3 WSSV-infected shrimp with the same body weights were cultured with one African sharptooth catfish of different body weights. After 14 days in culture, the ponds cocultured with one catfish weighing 0.25kg, 0.5 kg, 0.75 kg, and 1.5 kg showed shrimp survival rates of 19%, 40.83%, 48.67%, and 55.5%, respectively. Nearly all dead shrimp were removed by fish in the ponds cocultured with catfish of bodyweights greater than 0.5 kg. This suggests the suitable body weight of co-cultured catfish to control WSS outbreak is 0.5kg. Furthermore, co-culturing of shrimp and fish can control the WSS outbreaks even when there are WSSV carriers in shrimp postlarva.

Supplementary Figure 16. Mathematical modeled threshold parameters of using fish to control WSS spreads and experimental verification

Body weight of shrimp (gram)	Number of expt.	Body weight of fish (kg)	Number of healthy shrimps	Success* (No. of infected shrimps)	Failure** (No. of infected shrimps)	Threshold of simulation***
1.3	1	1-kg	750	3		182
	2	1-kg	750	6		
	3	1-kg	750	9		
	4	1-kg	750	12		
	5	1-kg	750	15		
	6	1-kg	750	18		
	7	1-kg	750	21		
2.5	1	1-kg	750	10		88
	2	1-kg	750	20		
	3	1-kg	750	30		
	4	1-kg	750	40		
	5	1-kg	750	50		
	6	1-kg	750	60		
	7	1-kg	750	70		
5.0	1	1-kg	750	50		53
	2	1-kg	750		70	
	3	1-kg	750		90	
	4	1-kg	750		110	
	5	1-kg	750		120	
	6	1-kg	750		130	
	7	1-kg	750		140	
7.8	1	1-kg	750	30		32
	2	1-kg	750		40	
	3	1-kg	750		50	
	4	1-kg	750		60	

Note:

* the initial number of WSSV-infected shrimp that can be successfully controlled by coculturing one 1-kg grass carp.

** the initial number of WSSV-infected shrimp that fail to be controlled by coculturing one 1-kg grass carp.

*** the max initial number of infected shrimps, which can be successfully controlled by coculturing one 1-kg grass carp, derived from Model 3

Supplementary Table 17. The number of cocultured grass carp and the occurrence rate of WSS

Number of fish	Occurrence rate of WSS
0	80%
45	80%
150	40%
225	40%
300	0
450	0
600	0
750	0

Supplementary Table 18. The number of cocultured African sharptooth catfish and the occurrence rate of WSS

Number of fish	Occurrence rate of WSS
0	80%
150	80%
300	80%
450	60%
600	0
750	0
900	0

Supplementary Table 19. Effectiveness of the control of WSSV by grass carp in *L. vannamei* production at a farm in Maoming, Guangdong Province, China (Farm 1)

Zone-Year	Area (ha)	Total Pond	Success Pond		Yield (kg/ha)	Success (%)	
			Area (ha)	Pond	Mean ± S.D.	Area Ratio	Pond Ratio
A-2011	6.03	18	5.70	17	7,332 ± 2,059	94.53	94
B-2011	11.03	28	2.73	8	1,844 ± 1034	24.75	29
A-2012	6.03	18	1.50	6	1,953 ± 1,188	24.88	33
B-2012	11.03	28	11.03	28	8,587 ± 1,655	100.00	100

Supplementary Table 20. Effectiveness of the control of WSSV by African sharptooth catfish in *L. vannamei* production at a farm in Qinzhou, Guangxi Province, China (Farm 2)

Zone-Year	Area (ha)	Total Pond	Success		Yield (kg/ha)	Success (%)	
			Area (ha)	Pond	Mean ± S.D.	Area Ratio	Pond Ratio
A-2011	21.20	38	21.20	38	8,730 ± 1,187	100.00	100
B-2011	67.00	57	5.33	4	1,450 ± 820	7.96	7
A-2012	21.20	38	21.20	38	9,628 ± 1,471	100.00	100
B1-2012	27.00	25	27.00	25	6,375 ± 1,000	100.00	100
B2-2012	40.00	32	4.36	3	1,900 ± 1500	10.90	9

Supplementary Table 21. Effectiveness of the control of WSSV by brown-marbled grouper (*Epinephelus fuscoguttatus*) in *P. monodon* production at a farm in Changjiang, Hainan Province, China (Farm 3)

Zone-Year	Area (ha)	Total Pond	Success Pond		Yield (kg/ha)	Success (%)	
			Area (ha)	Pond	Mean ± S.D.	Area Ratio	Pond Ratio
A-2013	1.6	6	1.6	6	6,395 ± 427	100	100
B-2013	0.8	3	0	0	1,622 ± 418	0	0
A-2014	1.6	6	1.6	6	6,440 ± 447	100	100
B-2014	0.8	3	0	0	1,960 ± 373	0	0

Supplementary Table 22. Effectiveness of the control of WSSV by branded goby (*Chaeturichthys stigmatias*) in *M. japonica* production at a farm in Qingdao, Shandong Province, China (Farm 4)

Zone-Year	Area (ha)	Total Pond	Success Pond		Yield (kg/ha)	Success (%)	
			Area (ha)	Pond	Mean ± S.D.	Area Ratio	Pond Ratio
A-2013	13.4	10	13.4	10	1,089 ± 50	100	100
B-2013	6.7	5	1.34	1	494 ± 48	20	20
A-2014	13.4	10	13.4	10	1,121 ± 48	100	100
B-2014	6.7	5	0	0	407 ± 16	0	0

Supplementary Table 23. The yield of ponds at farms' association in Nansha, Guangdong Province, China in 2015

Farmer	Area (ha)	Total Pond	Yield of Shrimps (kg)	Yield of Fish (kg)
1	3	1	4,050	6,000
2	3	1	4,595	6,000
3	3	1	3,625	6,450
4	3	1	3,580	6,550
5	3	1	3,260	6,200
6	3	1	3,285	6,400
7	3	1	1,625	-
8	3	1	1,995	-
9	3	1	1,710	-
10	3	1	1,595	-
11	3	1	1,655	-
12	3	1	1,610	-

Note: Farmer 1 to 6 adopted polyculture system in 2015. Six farmers (Farmer 7 to 12) who did not adopt the polyculture system were randomly selected.

Supplementary Table 24. The yield of ponds at farms' association in Tanghai, Guangdong Province, China in 2014 and 2015

2014

Farmer	Area (ha)	Total Pond	Yield of Shrimps (kg)	Yield of Fish (kg)
1	5	1	143	-
2	5	1	164	-
3	5	1	194	-
4	5	1	185	-
5	5	1	174	-
6	5	1	169	-
7	5	1	158	-
8	5	1	162	-
9	5	1	193	-
10	5	1	207	-

2015

Farmer	Area (ha)	Total Pond	Yield of Shrimps (kg)	Yield of Fish (kg)
1	5	1	1,201	516
2	5	1	1,022	644
3	5	1	1,097	653
4	5	1	933	527
5	5	1	1,269	659
6	5	1	1,075	370
7	5	1	1,134	459
8	5	1	1,403	776
9	5	1	1,216	806
10	5	1	1,239	784

Supplementary Table 25. The estimation of a, b, c_1 and c_2 under different weight w

w	a	b	c_1	c_2
2.0g	4.7321	0.5226	0.9798	1.1052
6.1g	5.2799	0.4666	1.0753	1.1569
8.1g	4.4270	0.4742	0.8950	1.1849
