# Supplementary material for: A convenient polyculture system that controls a shrimp viral disease with a high transmission rate
Source: Commun Biol. 2021 Nov 11;4:1276. doi: 10.1038/s42003-021-02800-z (PMC8585955; doi:10.1038/s42003-021-02800-z)
Supplement: Supplementary file 3 — Description of Additional Supplementary Files [file 42003_2021_2800_MOESM3_ESM.pdf]

Description of Additional Supplementary Files

File name: Supplementary Data 1

Description: Shrimp productions with or without co-cultured fishes at the farm in Maoming, Guangdong Province, China (Farm 1) from 2013 to 2019.
